# Agreement between hospital and primary care on diagnostic labeling for COPD and heart failure in Toronto, Canada: a cross-sectional observational study

**DOI:** 10.1038/s41533-018-0076-8

**Published:** 2018-03-09

**Authors:** Michelle Greiver, Frank Sullivan, Sumeet Kalia, Babak Aliarzadeh, Deepak Sharma, Steven Bernard, Christopher Meaney, Rahim Moineddin, David Eisen, Navid Rahman, Tony D’Urzo

**Affiliations:** 1 Department of Family and Community Medicine, Faculty of Medicine, University of Toronto, Toronto, Canada; 2 North York Family Health Team, North York, Canada; 3 Department of Family and Community Medicine, North York General Hospital, North York, Canada; 4 Institute for Clinical Evaluative Sciences, Toronto, Canada; 5 Medical School, University of St Andrews, St Andrews, UK; 6 North York General Hospital, North York, Canada

## Abstract

Patients with chronic obstructive pulmonary disease (COPD) or heart failure (HF) are frequently cared for in hospital and in primary care settings. We studied labeling agreement for COPD and HF for patients seen in both settings in Toronto, Canada. This was a retrospective observational study using linked hospital-primary care electronic data from 70 family physicians. Patients were 20 years of age or more and had at least one visit in both settings between 1 January 2012 and 31 December 2014. We recorded labeling concordance and associations with clinical factors. We used capture-recapture models to estimate the size of the populations. COPD concordance was 34%; the odds ratios (ORs) of concordance increased with aging (OR 1.84 for age 75+ vs. <65, 95% CI 0.92–3.69) and more inpatient admissions (OR 2.89 for 3+ visits vs. 0 visits, 95% CI 1.59–5.26). HF concordance was 33%; the ORs of concordance decreased with aging (OR 0.39 for 75+ vs. <65, 95% CI 0.18–0.86) and increased with more admissions (OR = 2.39; 95% CI 1.33–4.30 for 3+ visits vs. 0 visits). Based on capture-recapture models, 21–24% additional patients with COPD and 18–20% additional patients with HF did not have a label in either setting. The primary care prevalence was estimated as 748 COPD patients and 834 HF patients per 100,000 enrolled adult patients. Agreement levels for COPD and HF were low and labeling was incomplete. Further research is needed to improve labeling for these conditions.

## Diagnostic labeling: accuracy needed for serious health conditions

Improving diagnostic labeling across healthcare providers would help develop a more robust database to improve care and inform research. Patient diagnoses for serious conditions, such as heart failure (HF) and chronic obstructive pulmonary disease (COPD), should be documented across care providers, regardless of whether a patient is seen in hospital or local surgery. Consistent labeling of patient charts across providers improves patient services and informs research into clinical practice. Michelle Greiver at North York General Hospital in Toronto, Canada, and co-workers uncovered significant disagreement between a Toronto hospital and its associated primary care providers on labeling of patients with HF or COPD, with the two settings agreeing on labels for a third of patients. Around one fifth of patients had no diagnostic label in either setting, despite suffering from one or other condition.

## Introduction

A foundational activity for the assessment of quality of care is correctly labeling patients that have a health condition of interest so that patient cohorts can be generated.^1^ Quality of care can then be measured, monitored, and improved for this cohort. Patients with high-risk conditions such as heart failure (HF) or chronic obstructive pulmonary disease (COPD) often receive care in both primary care and hospital settings. Exacerbations of COPD and HF are leading causes of hospital admissions in many parts of the world^2–4^ and there is increasing focus on strategies to reverse these trends. Upon discharge from hospital these patients are often advised to follow-up with their primary care physician or health care provider. Understanding how patient diagnoses are documented in these different settings has important clinical and research implications. For example, estimating levels of agreement between diagnostic labeling in primary care practices and hospitals that share the care of patients may facilitate patient management and provide clarity on the state of research preparedness of respective databases, including the size of the populations of interest.

Presently, integrated care systems do not exist in Canada’s largest province, Ontario. However, the province has geographically based referral patterns and informal physician networks.^5^ That is, primary care physicians have high “loyalty” and tend to refer to local colleagues and local hospitals. This loyalty has recently been estimated to be about 70%,^5^ suggesting that most of the clinical data on both primary and inpatient care for majority of patients exists in combined local systems.

In Toronto, Canada, the North York General Hospital (NYGH) and its affiliated primary care physician community have recently co-created a hospital primary care analytical database, the Health Databank Collaborative (HDC).^6^ This database contains linked clinical data from NYGH and data from the primary care Electronic Medical Records (EMRs) of consenting family physicians, extracted and managed through work done by the UTOPIAN Primary Care Research Network, 1 of 11 networks participating in the Canadian Primary Care Sentinel Surveillance Network, CPCSSN.^7^

Our objectives were to generate cohorts of patients seen in both settings labeled as having HF or COPD, to estimate agreement on recorded labels of COPD or HF, to determine patient factors associated with agreement on labeling, and to estimate the size of the populations of interest.

## Results

Seventy primary care physicians affiliated with NYGH contributed EMR data to HDC during this study. There were 101,501 patients age 20 or more as of 31 December 2014 enrolled with the practices of these physicians. Patient characteristics are shown in Tables 1a and 1b. The odds ratios and adjusted probabilities of having a concordant diagnostic label for COPD and HF are shown in Tables 2 and 3, respectively.

**Table 1a:** Patient characteristics according to setting with chronic obstructive pulmonary disease labeling

	Patients labeled only in primary care		Patients labeled only in hospital		Patients labeled in both settings		Total
	*N*	Row percentage (%)	*N*	Row percentage (%)	*N*	Row percentage (%)	*N*
Age range (years)							
<65	52	62.7	17	20.5	14	16.9	83
65–74	38	40.9	28	30.1	27	29.0	93
75+	122	30.3	126	31.3	154	38.3	402
Deceased							
No	200	41.4	136	28.2	147	30.4	483
Yes	12	12.6	35	36.8	48	50.5	95
Gender							
F	125	36.7	102	29.9	114	33.4	341
M	87	36.7	69	29.1	81	34.2	237
No. of co-morbidities							
0–1	31	21.8	89	62.7	22	15.5	142
2	63	41.7	41	27.2	47	31.1	151
3+	118	41.4	41	14.4	126	44.2	285
Income quintiles							
1 (lowest income)	18	33.3	8	14.8	28	51.9	54
2	25	43.1	14	24.1	19	32.8	58
3	28	35.0	23	28.8	29	36.3	80
4	54	46.6	28	24.1	34	29.3	116
5 (highest income)	60	39.7	34	22.5	57	37.7	151
Missing	27	22.7	64	53.8	28	23.5	119
No. of visits to emergency department^a^							
0	29	21.8	59	44.4	45	33.8	133
1	103	49.0	49	23.3	58	27.6	210
2	32	39.5	15	18.5	34	42.0	81
3+	48	31.2	48	31.2	58	37.7	154
No. of encounters with primary care physician^a^							
1–2	5	21.7	11	47.8	7	30.4	23
3–9	24	27.3	35	39.8	29	33.0	88
10+	183	39.2	125	26.8	159	34.0	467
No. of inpatient admissions^a^							
0	132	61.4	36	16.7	47	21.9	215
1	55	30.7	59	33.0	65	36.3	179
2	12	14.8	35	43.2	34	42.0	81
3+	13	12.6	41	39.8	49	47.6	103
Total	212	36.7	171	29.6	195	33.7	578

^a^During the 3-year period of interest (1 January 2012 to 31 December 2014)

**Table 1b:** Patient characteristics according to setting with heart failure labeling

	Patients labeled only in primary care		Patients labeled only in hospital		Patients labeled in both settings		Total
	*N*	Percentage (%)	*N*	Percentage (%)	*N*	Percentage (%)	*N*
Age range (years)							
<65	8	21.1	13	34.2	17	44.7	38
65–74	21	28.4	25	33.8	28	37.8	74
75+	104	18.0	295	50.9	180	31.1	579
Deceased							
No	113	21.4	256	48.5	159	30.1	528
Yes	20	12.3	77	47.2	66	40.5	163
Gender							
F	83	19.3	217	50.5	130	30.2	430
M	50	19.2	116	44.4	95	36.4	261
No. of co-morbidities							
0–1	29	11.2	176	67.7	55	21.2	260
2	42	21.3	81	41.1	74	37.6	197
3+	62	26.5	76	32.5	96	41.0	234
Income quintiles							
1 (lowest income)	13	21.0	23	37.1	26	41.9	62
2	15	20.8	25	34.7	32	44.4	72
3	25	27.2	32	34.8	35	38.0	92
4	27	22.1	53	43.4	42	34.4	122
5 (highest income)	34	21.7	57	36.3	66	42.0	157
Missing	19	10.2	143	76.9	24	12.9	186
No. of visits to emergency department^a^							
0	21	9.7	121	55.8	75	34.6	217
1	57	28.4	86	42.8	58	28.9	201
2	18	15.5	54	46.6	44	37.9	116
3+	37	23.6	72	45.9	48	30.6	157
No. of encounters with primary care physician^a^							
1–2	1	1.9	41	75.9	12	22.2	54
10+	118	22.7	221	42.5	181	34.8	520
3–9	14	12.0	71	60.7	32	27.4	117
No. of inpatient admissions^a^							
0	68	48.9	36	25.9	35	25.2	139
1	46	18.1	122	48.0	86	33.9	254
2	10	7.9	73	57.9	43	34.1	126
3+	9	5.2	102	59.3	61	35.5	172
Total	133	19.2	333	48.2	225	32.6	691

^a^During the 3-year period of interest (1 January 2012 to 31 December 2014)

**Table 2:** Odds ratios of having concordant labeling (label present in both settings) for COPD and heart failure

			COPD	Heart failure
Effect	Index group	Reference group	Odds ratio (95% confidence interval)	Odds ratio (95% confidence interval)
Age range (years)	65–74	<65	1.37 (0.62–3.05)	0.47 (0.19–1.17)
	75+	<65	1.84 (0.92–3.69)	**0.39 (0.18–0.86)**
Gender	F	M	0.92 (0.62–1.38)	0.72 (0.49–1.05)
Deceased	Yes	No	**1.9 (1.12–3.23)**	**1.99 (1.29–3.08)**
No. of co-morbidities	2	0–1	**2.95 (1.49–5.83)**	**1.76 (1.08–2.88)**
	3+	0–1	**4.6 (2.41–8.76)**	**2.04 (1.26–3.29)**
Income quintiles	2	1	**0.35 (0.15–0.82)**	**1.33 (0.62–2.86)**
	3	1	0.51 (0.23–1.11)	1.07 (0.52–2.21)
	4	1	**0.35 (0.17–0.74)**	0.73 (0.37–1.47)
	5	1	0.5 (0.25–1.02)	1.13 (0.58–2.21)
No. of ED visits^a^	1	0	1.05 (0.58–1.9)	0.68 (0.41–1.15)
	2	0	1.48 (0.75–2.94)	1.13 (0.65–1.99)
	3+	0	1.12 (0.62–2.02)	0.69 (0.4–1.18)
No. of inpatient visits^a^	1	0	**1.91 (1.11–3.3)**	**1.95 (1.13–3.39)**
	2	0	**2.47 (1.29–4.73)**	**2.05 (1.08–3.89)**
	3+	0	**2.89 (1.59–5.26)**	**2.39 (1.33–4.3)**
No. of primary care visits^a^	3 to 9	1 to 2	0.84 (0.26–2.67)	1.02 (0.42–2.47)
	10+	1 to 2	0.58 (0.19–1.72)	1.23 (0.54–2.8)

*COPD* chronic obstructive pulmonary disease, *ED* emergency department

Significant results have been bolded

^a^During the 3-year period of interest (1 January 2012 to 31 December 2014)

**Table 3:** Adjusted probabilities of having concordant labeling (label present in both settings) for COPD and heart failure

		COPD	Heart failure
Effect	Value	Adjusted probability (95% confidence interval)	Adjusted probability (95% confidence interval)
Age range (years)	<65	0.32 (0.18–0.5)	0.59 (0.39–0.76)
	65–74	0.39 (0.25–0.55)	0.40 (0.26–0.56)
	75+	0.46 (0.35–0.57)	0.36 (0.27–0.45)
Gender	F	0.38 (0.27–0.5)	0.41 (0.3–0.53)
	M	0.40 (0.28–0.53)	0.49 (0.37–0.62)
Deceased	Yes	0.47 (0.32–0.62)	0.53 (0.4–0.67)
	No	0.31 (0.22–0.43)	0.37 (0.27–0.48)
No. of co-morbidities	0–1	0.21 (0.13–0.32)	0.35 (0.24–0.47)
	2	0.44 (0.3–0.59)	0.48 (0.35–0.62)
	3+	0.55 (0.41–0.69)	0.52 (0.38–0.65)
Income quintiles	1	0.58 (0.4–0.74)	0.48 (0.31–0.65)
	2	0.32 (0.19–0.5)	0.55 (0.39–0.7)
	3	0.41 (0.26–0.57)	0.50 (0.34–0.65)
	4	0.32 (0.2–0.47)	0.40 (0.27–0.55)
	5	0.41 (0.28–0.55)	0.51 (0.38–0.65)
No. of ED visits^a^	0	0.36 (0.24–0.5)	0.49 (0.36–0.61)
	1	0.37 (0.24–0.51)	0.39 (0.27–0.53)
	2	0.45 (0.29–0.62)	0.52 (0.37–0.67)
	3+	0.38 (0.26–0.52)	0.40 (0.26–0.55)
No. of inpatient visits^a^	0	0.25 (0.16–0.37)	0.32 (0.20–0.46)
	1	0.39 (0.26–0.53)	0.47 (0.35–0.6)
	2	0.45 (0.3–0.61)	0.49 (0.34–0.64)
	3+	0.49 (0.33–0.64)	0.53 (0.39–0.66)
No. of primary care visits^a^	1–2	0.45 (0.21–0.7)	0.43 (0.24–0.64)
	3–9	0.40 (0.27–0.55)	0.43 (0.3–0.58)
	10+	0.32 (0.24–0.41)	0.48 (0.38–0.58)

*COPD* chronic obstructive pulmonary disease, *ED* emergency department

^a^During the 3-year period of interest (1 January 2012 to 31 December 2014)

### COPD

Five hundred seventy-eight patients had charts labeled with a diagnosis of COPD in at least one setting. 59% were women; the mean age was 77.6 years and the mean number of co-morbidities was 2.49. 36% of these patients were labeled as having COPD only in primary care, 30% were labeled only in the hospital and 34% were concordant (the two settings agreed on labeling). Of those concordant, 128 (65%) were initially labeled as having a diagnosis of COPD in primary care and 67 (35%) were initially labeled with COPD in the hospital.

The odds ratios and adjusted probabilities of concordant labeling were higher with increasing age (OR 1.84 for patients age 75 years or more compared to <65, 95% CI 0.92–3.69), greater number of co-morbidities (OR 4.6 for 3 or more co-morbidities compared to 0 or 1, 95% CI 2.4–8.8), and higher number of inpatient admissions (OR 2.9 for 3 or more inpatient admissions compared to none, 95% CI 1.6–5.3). The most socioeconomically deprived patients were more likely to have concordant labeling, although this effect was not statistically significant (OR 0.50 for least deprived vs. most deprived, 95% CI 0.25–1.02). There were no significant differences by patient gender, number of emergency department visits, or number of primary care encounters.

37% of patients with COPD were seen in the ED only (no inpatient admissions). 21.9% of those had concordant labeling as shown in Tables 1a and 1b. The adjusted probability of concordance for patients with ED visits only was 25% (95% CI 16–37%) as shown in Table 2.

### Heart failure

Six hundred ninty-one patients had charts labeled with a diagnosis of HF in at least one setting. 62% were women; the mean age was 83 years and the mean number of co-morbidities was 2.0. 19% were labeled as having HF only in primary care, 48% were labeled only in the hospital and 33% were concordant (the two settings agreed on labeling). Of those concordant, 88 (39%) were initially labeled as having a diagnosis of HF in primary care and 137 (61%) were initially labeled in the hospital.

The odds ratios and adjusted probabilities of patients having a label of HF in both settings decreased as age increased (OR 0.39 for patients age 75 years or more compared to <65, 95% CI 0.18–0.86). Patients that died during the period of observation were more likely to have agreement on diagnostic labeling (OR = 1.99, 95% CI 1.29–3.08). An increasing number of co-morbidities also increased the likelihood of concordance (OR = 2.04 for 3 or more co-morbidities vs. none; 95% CI 1.26–3.09), as were more inpatient visits (OR = 2.39; 95% CI 1.33–4.30 for 3+ visits vs. 0 visits). There were no significant differences by patient gender, income quintiles, number of emergency department visits, or number of primary care encounters.

20% of patients with HF were seen in the ED only (no inpatient admissions); 25.2% of those had concordant labeling as shown in Tables 1a and 1b. The adjusted probability of concordance for patients with ED visits only was 32% (95% CI 20–46%), as shown in Table 2.

### Capture-recapture models

**Fig. 1:**
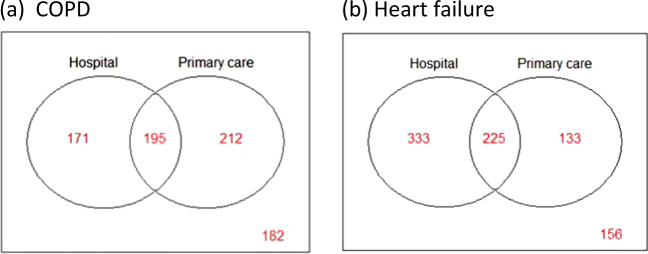
Venn diagrams for number of patients labeled for COPD and heart failure per site, using capture-recapture models: **a** COPD, One hundred seventy-one patients were labeled only in the hospital, 212 were labeled only in primary care, and 195 in both. One hundred eighty-two patients were not labeled in either setting. **b** Heart failure, Three hundred thirty-three patients were labeled only in the hospital, 133 were labeled only in primary care, and 225 in both. One hundred fifty-six patients were not labeled in either setting

Using capture-recapture models, we determined that 21–24% additional patients with COPD and 18–20% additional patients with HF did not have the condition labeled in their chart in either setting. The data are presented in Tables 4a and 4b and Fig. 1. After adjustment for patient characteristics, the population size was estimated to be 760 patients (95% CI 695–837) with COPD and 847 patients (95% CI 787–918) with HF. Given that there were 101,501 patients enrolled to the primary care physicians in this cohort, we expect to find 748 COPD patients per 100,000 enrolled adult patients and 834 HF patients per 100,000 enrolled adult patients.

**Table 4a:** Proportions of patients with a label of COPD captured in different settings, estimated additional proportion of missing patients and estimated population size

Model	Patients labeled in hospital	Patients labeled in primary care	Patients labeled in both	Additional patients not labeled in either setting	Estimated population size (95% confidence interval)
COPD = no covariate	30%	37%	34%	24% (19–30%)	764 (710–827)
COPD = age	30%	36%	34%	24% (19–30%)	763 (709–826)
COPD = age + sex	32%	34%	34%	24% (18–30%)	763 (708–828)
COPD = age + sex + income quintile	29%	34%	37%	21% (15–27%)	734 (681–796)
COPD = age + sex + income quintile + comorbidities	32%	34%	34%	24% (17–31%)	760 (695–837)

**Table 4b:** Proportions of patients with a label of heart failure captured in different settings, estimated additional proportion of missing patients and estimated population size

Model	Patients labeled in hospital	Patients labeled in primary care	Patients labeled in both	Additional patients not labeled in either setting	Estimated population size (95% confidence interval)
HF = no covariate	46%	19%	35%	20% (14–26%)	859 (799–929)
HF = age	46%	19%	35%	20% (14– 26%)	860 (799–930)
HF = age + sex	36%	25%	36%	20% (14–27%)	867 (800–946)
HF = age + sex + income quintile	34%	26%	39%	19% (12–25%)	849 (787–923)
HF = age + sex + income quintile + comorbidities	34%	26%	39%	18% (12–25%)	847 (787–918)

*COPD* chronic obstructive pulmonary disease, *HF* heart failure

## Discussion

We found significant disagreement between hospital and primary care records on labeling for COPD and HF as the two settings agreed on only a third of patients. About a fifth of all patients had no diagnostic labels in either setting. Agreement on a diagnostic label was more likely when there were more inpatient admissions but not when there were more primary care visits. The estimated prevalence of COPD or HF associated with hospitalization in primary care practices was slightly less than 1% for each condition.

There is scant information on labeling agreement between hospital and primary care for chronic conditions with high impact on health; one study in Spain found low concordance for a number of conditions, including COPD and HF.^8^ A study in Scotland found low levels of concordance for incident myocardial infarcts, strokes, and ischemic heart disease.^9^ The lack of agreement on diagnostic labels in our study is surprising, given the fact that the two conditions we studied are associated with significant morbidity and mortality and are leading causes of hospital admission and re-admissions.^2–4^

Misclassifications of COPD and HF (both under-diagnosis and overdiagnosis) are common.^10–12^

Chart audits for the presence of appropriate testing and diagnostic criteria would be needed in both settings to determine diagnostic accuracy of in this study. This would include spirometry for COPD^13^ and echocardiography and brain natriuretic peptide for HF.^12,14^ We therefore make no inferences about the accuracy of labeling. It would be reasonable to assume that there are errors in both settings and this would at least partially explain the lack of agreement.

The prevalence of COPD in Canada has been estimated at 4% using self-reports, which underestimate COPD.^15^ Recent spirometry testing for a random sample of the population as part of the Canadian Health Measures Survey found a prevalence of 17% for COPD.^16^ The prevalence of moderate to severe COPD (GOLD III and IV) was 1%.^16^ We report an estimated prevalence of 0.9% in this population of patients with hospital admissions. We are not able to determine from our study whether this difference is due to under-diagnosis or to our population having more severe disease. Under-diagnosis of COPD is a common finding in primary care,^17,18^ and this might in part be related to lack of access to spirometry in primary care.^19^

It is relevant to note that even among patients admitted to hospital with a diagnosis of COPD, less than 10% of such patients undergo confirmatory spirometry and about one-third do not demonstrate spirometric features compatible with COPD.^20^ These data highlight that factors affecting diagnostic labeling may include availability of objective data to confirm COPD diagnosis as well as practices utilized in both the hospital and community clinic settings which drive how data are entered into medical records.

The HF prevalence of 0.8% in our study is comparable to the self-reported prevalence of HF in Canada of 1%.^21^ Yearly rates of hospital admission for HF in Canada have been decreasing and were 204 per 100,000 population in 2014.^22^ However, a recent study found that 83.1% of patients followed with HF in the community have had at least one hospitalization in a 5-year period, with the majority of reasons (61.9%) being non-cardiovascular.^23^

More patients were labeled with COPD only in the community rather than only in the hospital. Many cases of COPD are mild (GOLD I and II) and may not be perceived as impacting morbidity in the hospital but could be labeled in the community due to case finding for patients who smoke or have clinical features such as chronic cough. The opposite was present for HF, with 48% of patients labeled only in the hospital, even though they were seen by their family physician after a hospitalization. Both COPD and HF are Ambulatory Care Sensitive Conditions, meaning that optimal care in the community can reduce hospitalizations and emergency department visits.^24^ Appropriate diagnostic labeling of patient charts is needed to monitor quality of care.^1^ Reasons for lack of labeling in the community following hospitalization could include poor communication between hospital and primary care^25^ or disagreement about the diagnosis. Reducing this gap in concordance is important it terms of promoting a robust primary care data base for research.

Concordance for both conditions increased with an increasing number of hospitalizations during the 3-year period, perhaps reflecting more opportunities for reports mentioning the conditions being forwarded from the hospital to primary care. Older age was associated with greater concordance for COPD but the opposite for HF; this unexpected finding should be confirmed and would benefit from further study. Some factors that might have influenced our findings include the fact that approximately half of hospital re-admissions in patients with HF are related to co-morbidities, polypharmacy, and other conditions associated with HF.^26^

Our patients with HF were older than patients with COPD (77 vs. 83 years). Furthermore, we noted that there were more female patients among our population of HF patients. Older HF patients are more likely to be female.^26^ These patients also tend to have higher rates of non-cardiovascular conditions,^26^ which may drive patients to seek medical attention and influence diagnostic labeling in both the primary and hospital settings.

It is important to mention that we did not evaluate concordance among those patients with a diagnosis of both HF and COPD. Among individuals with HF the prevalence of COPD ranges between 20 and 32%, and reports suggest that 10% of hospitalized HF patients also suffer from COPD.^27^ Conversely, HF is prevalent in more than 20% of patients with COPD.^28,29^ It was beyond the scope of this study to understand how concomitant illness with COPD and HF might influence concordance rates in the two settings we studied.

Understanding the concordance of this particularly high-risk population between the two settings and confirmation of diagnosis by echocardiography and spirometry would be advantageous in the overall care of such individuals.

### Limitations

The study had several strengths. It reflected data from routine clinical care for patients with COPD and HF in community-based primary care and hospital care. Data were extracted from several different EMR platforms, accounting for a variety of EMR-specific data entry processes for diagnostic labeling by clinicians. Despite these strengths, this study includes several shortcomings. This was a convenience sample of primary care practices that contributed EMR data to UTOPIAN, rather than a random sample. Correction of an inaccurate diagnosis is another explanation for some of the discrepancy. For example, a chesty smoker may be coded as “COPD” by a physician in the ED and subsequently corrected by the family physician after spirometry. A breathless patient coded by a family physician as HF may be corrected after testing during a hospital admission. However, we note that in Canadian family practices, adding a health condition to the summary health profile is not a trivial activity, as this is considered the “master list” for significant problems in the health record, and is recognized as such by regulatory authorities.^30^

We only counted hospital admissions and ED visits; there are several other reasons for a visit to the hospital, including diagnostic imaging. We would not necessarily expect complete labeling for major health conditions if the patient presented for imaging only. It is reasonable, however, to expect major conditions, such as COPD or HF, to be labeled in a patient’s chart during a hospital admission.

Patients may present to ED with minor conditions; coding for COPD or HF may not have been entered in those cases. We were unable to exclude visits for minor conditions from the dataset and provided results excluding patients seen only in ED. In a random audit of 100 patients with COPD and HF for this study, only three ED visits with COPD and two ED visits with HF were not followed by an admission.

We did not collect several variables relevant to the diagnosis of COPD or HF, as these were not available in the HDC database. These variables include echocardiograms and spirometry results. The data were from a single hospital, and do not reflect information on patients admitted to other hospitals in the area. However, regional loyalty in our setting is reasonably high at an estimated 70%, reflecting the share of patients with COPD or HF from primary care physicians seen at the hospital.^5^ We studied labeling concordance, which would not be impacted by admission elsewhere. All family physicians in this study were affiliated with NYGH.

### Conclusions

We found low rates of labeling agreement for COPD and HF between a hospital and its primary care community as well as missing labels in both settings. This points to opportunities to improve the documentation of these high-risk, high-cost conditions. Further research is needed to understand and explore factors that influence diagnostic labeling and agreement. Identifying strategies to improve diagnostic labeling between the hospital and community clinics may also serve to develop robust data bases which can be used to promote patient care and collaborative research initiatives.

## Methods

### Study design

This was a cross-sectional retrospective observational study using data from the NYGH HDC database; we applied the STROBE checklist for reporting observational studies.^31^

### Data sources

EMR data routinely entered in primary care charts were used for this study. These data were extracted and managed through work done by UTOPIAN, the University of Toronto Practice-Based Research Network. UTOPIAN is one of 12 networks participating in the Canadian Primary Care Sentinel Surveillance Network, CPCSSN.^7^ CPCSSN is Canada’s largest primary care EMR-based research and surveillance database; all contributing networks use similar data extraction and management processes and these have been previously described.^7^ Briefly, consenting family physicians and other primary care providers contribute de-identified EMR data to the UTOPIAN data repository; posters informing patients about the study are present in the waiting rooms of participating practices, patients can opt-out if they choose to do so.^7,32^

The primary care data were linked to the hospital’s database using an encrypted identifier for each patient.^6^ The linked data were analyzed.

### Patient population

**Fig. 2:**
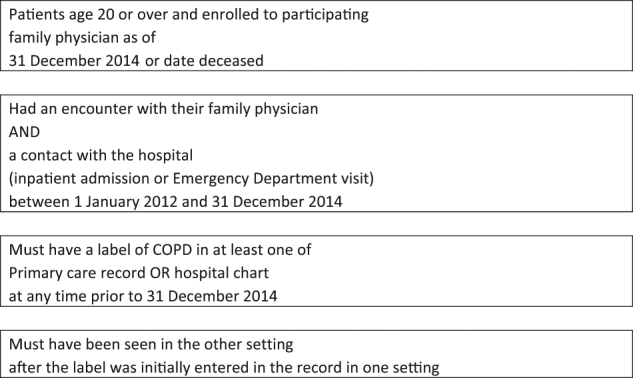
Flow diagram for cohort generation, COPD

The population of interest consisted of patients age 20 or more as of 31 December 2014 or as of date deceased, seen at least once in both settings (hospital and primary care) during a 3-year period (1 January 2012 to 31 December 2014). Patients were enrolled to a family physician contributing data to the HDC. There must have been a diagnostic label of COPD or HF recorded in at least one setting at any time prior to 31 December 2014. There must have been at least one visit to the primary care physician following the hospital visit where HF or COPD had initially been recorded, or one Hospital visit where there was a pre-existing HF or COPD diagnosis in the primary care record. The generation of the cohort for COPD is described in Fig. 2. A similar process was used for HF.

For the hospital, standardized information on acute inpatient care and emergency care is extracted from each chart and recorded using ICD10 codes in the Canadian Institute for Health Information Discharge Abstract Database (CIHI DAD)^33^ and CIHI National Ambulatory Care Reporting System (NACRS).^34^ Trained auditors abstract data from the chart, using a standardized manual, and record findings as structured data. The CIHI diagnostic types used were the Most Responsible Diagnosis and pre-admit comorbidity.^33^

We used ICD-10-CA codes for HF consistent with those in CIHI DAD/NACRS and recommended as indicating HF by the recent Health Quality Ontario Quality-Based Procedures: I50.x, I25.5, I40.x, I41.x, I42.x, I43.x.^35^ For COPD, we used ICD-10-CA J41-J44.^36^

For primary care, we used data present in the summative health profile of the chart. In Canada, provincial licensing organizations recommend using the summative health profile as a standard area for recording the presence of important chronic conditions in primary care.^30,37^ We included patients with a coded diagnosis for HF (ICD9 428) or COPD (ICD9 491, 492, 496) or free text indicating either condition. A similar approach was used to validate the CPCSSN case definitions.^38^ Validation studies report good to excellent sensitivity and specificity for ICD9 codes 491, 492, and 496 for COPD^39^ and 498 for HF.^40^ CPCSSN has a coding tool that is applied when free text for diagnosis is present in the health profile and converts free text to ICD9 codes; we used this coding tool. In summary, any entry containing COPD, CHRONIC BRONCHITIS, EMPHYSEMA, CHRONIC AIRWAY OBSTRUCTION, CHRONIC OBSTRUCTIVE PULMONARY DISEASE, and any other keywords that indicate COPD were coded as COPD. HEART FAILURE, HEARTFAILURE, HT FAILURE, BIVENTRICULAR FAILURE, CARDIAC FAILURE, WEAK HEART, CHF, CONGESTIVE HEART DISEASE, VENTRICULAR FAILURE were used for HF. The tool excludes data where keywords indicate uncertainty about the diagnosis; these include “not”, “unlikely”, “unremarkable”, “absence of”, “check” before diagnosis keyword, or “?” before or after the diagnosis keyword.

The following data elements were extracted from the HDC database: patient age as of 31 December 2014 or date deceased, patient gender, presence of co-morbidities, socioeconomic quintiles, and health care utilization (number of primary care visits, hospital utilization). Co-morbidities were derived from eight CPCSSN validated disease case definition algorithms: diabetes, hypertension, osteoarthritis, depression, COPD, dementia, epilepsy, Parkinson’s disease.^38^ One of the conditions classified was COPD, so seven co-morbidities were measured for patients with COPD.

Hospital utilization was determined by the number of emergency department visits and inpatient admissions. If there was an emergency department visit followed by an inpatient admission, this was counted as one hospital inpatient admission.

We used geographically derived information to calculate income level.^41^ The Postal Code Conversion File, available from Statistics Canada, was used to link the six-character postal codes to the standard 2011 Census dissemination areas. Dissemination areas are small, stable parts of neighborhoods that include between 400 and 700 persons (http://www12.statcan.gc.ca/census-recensement/2011/ref/dict/geo021-eng.cfm). Subsequently, the Postal Code Conversion File was used to assign neighborhood income.^42^

### Statistical analysis

All statistical analyses were conducted using SAS software, version 9.4 (SAS Institute). We recorded whether labeling for COPD and HF was present in the hospital and primary care records. We examined the proportion of patients with concordant diagnostic labeling (where a label for each condition was present in both settings) and association between labeling concordance and clinical factors using random effects logistic regression model. We also used capture-recapture model to estimate the size of the patient population with COPD or HF that had been looked after in both hospital and primary care. Capture-recapture consists of taking a sample from a population, tagging each item sampled and placing Manuscript, all changes accepted them back. The population is then re-sampled and the tagged items are counted. The proportion of items tagged in the new sample reflects the proportion in the population and the size of the population can then be estimated. More details on capture-recapture model are provided in the Supplementary File.

This study was reviewed and appoved by the Research Ethics Board (REB) at the NYGH. UTOPIAN has received REB approval from the University of Toronto and from the NYGH for the collection of EMR data. All participating primary care providers have provided written informed consent for the collection and analysis of their EMR data.

### Data availability

The data that support the findings of this study are available from the NYGH’s Office of Research and Innovations. Requests for data may be made at http://www.nygh.on.ca/Default.aspx?cid=3157&lang=1. The data are not publicly available as they may contain patient-related information that could compromise patient privacy. Derived data supporting the findings of this study are available from the corresponding author upon request.

## Electronic supplementary material

Supplementary note
