# Supplementary material for: Agreement between hospital and primary care on diagnostic labeling for COPD and heart failure in Toronto, Canada: a cross-sectional observational study
Source: NPJ Prim Care Respir Med. 2018 Mar 9;28:9. doi: 10.1038/s41533-018-0076-8 (PMC5844864; doi:10.1038/s41533-018-0076-8)
Supplement: Supplementary file 1 — Supplementary note [file 41533_2018_76_MOESM1_ESM.docx]

**Appendix**

| **Capture-recapture model**  The random-effect multinomial model was fitted to estimate:  (1) the probability of patients being looked after in the hospital ($p_{1}$); these were patients with a diagnostic label recorded in the hospital  (2) the probability of patients being looked after in primary care ($p_{2}$); these were patients with a diagnostic label recorded in primary care  (3) the probability of patients being looked after in both settings ($p_{3}$); these were patients with a diagnostic label recorded in both settings.  The model proposed by Tilling and Sterne (44) allowed us to estimate the probability that patients had a condition of interest, were seen in both settings and had their diagnostic label missed by both ($p_{o}$). This probability is based on the maximum likelihood estimator and can be explicitly estimated as:  $p_{o}=\frac{1}{\left( 1+\frac{p_{3}}{p_{1}} \right)*\left( 1+\frac{p_{3}}{p_{2}} \right)}$    In the absence of any covariate adjustment, the capture-recapture model uses two important assumptions: (1) the capture probabilities of the two sources (i.e. list of patients in each setting) are independent, (2) the probability of being captured from each source is assumed to be the same for every subject. However, these assumptions are often violated in many epidemiology studies, contributing towards under or over estimation of the population size.(45-48) Hence, we analyzed this by stratifying the population estimate according to important patient characteristics: age range, gender, SES and co-morbidities. As part of the sensitivity analysis, we also presented the results with the inclusion of different combinations of patient characteristics. Assumption (1) and (2) are relaxed with the inclusion of patient characteristics in the random-effects multinomial regression model. |
| --- |
